# A proteomic analysis of acute leukemia cells treated with 9-hydroxyoctadecadienoic acid

**DOI:** 10.1186/s12944-016-0359-4

**Published:** 2016-11-10

**Authors:** Zhen Li, Bohong Chen, Ping Wang, Xin Li, Gaotai Cai, Wei Wei, Wenqi Dong

**Affiliations:** School of Laboratory Medicine and Biotechnology, Southern Medical University, Guangzhou, Guangdong Province 510515 People’s Republic of China

**Keywords:** 9s-hydroxy-octadecadienoic acid, Acute leukemia cells, Proteomics, Bioinformatics analysis

## Abstract

**Background:**

9s-hydroxy-octadecadienoic acid (9S-HOD), one of the natural products of linoleic acid oxygenation by 15-lipoxygenase (15-LOX), has been found to have anti-tumor properties in vitro and in vivo. The present study therefore investigated whether 9S-HOD affects acute leukemia HL-60 cells.

**Methods:**

The cytotoxicity of 9S-HOD in HL-60 with or without the presence of fetal bovine serum (FBS) in the culture media was tested using cell viability assays and flow cytometry. To explore the mechanism of its anti-tumor activity by 9S-HOD, we used a proteomic analysis to identify HL-60 cells protein profiles, based on two-dimensional gel electrophoresis (2-DE) and mass spectrometry (MS) identification.

**Results:**

9S-HOD exerted cytotoxicity efficacy and induced apoptosis in HL-60 cells, and the cytotoxicity was largely attenuated by the presence of FBS in culture media. The proteomic results revealed that 9S-HOD remarkably altered the abundance of 23 proteins that were involved in mRNA metabolic process, protein binding, DNA replication and apoptosis.

**Conclusions:**

Our results indicated that 9S-HOD exerts cytotoxicity in HL-60 cells by affecting several pathways.

## Background

Leukemia is one of the most common cancers in the world, especially the type of acute myeloid leukemia (AML). AML is characterized by the uncontrolled growth of abnormal white blood cells in the bone marrow and interfere with the production of normal blood cells [1]. Most current treatment strategies kill cancerous cells by chemotherapeutic agents with a number of severe toxic side-effects [2]. Therefore, the search for new anti-leukemia treatments has increased and natural products or their derivatives have been an important source due to their diverse activities [3].

Naturally occurring oxylipins, known as oxidized metabolites of long chain polyunsaturated fatty acids, have important roles in animals and plants for cell signaling and defense in response to attack from microbes or pathogens [4]. The hydroxyl-octadecadienoic acids (HODEs) are the major reaction products of linoleic acid oxygenation by 15-LOXenzyme, including 9-hydroxy-octadecadienoic acid (9-HOD) and 13-hydroxy-octadecadienoic acid (13-HOD) [5]. HODEs are well-known for its anti-inflammatory properties, particularly in cardiovascular and other inflammatory diseases [6]. Recently, studies have demonstrated that HODEs are associated with a number of cancers, revealing possible novel drugs for chemoprevention and/or treatment of a number of cancers prevention [7–9].

Multiple cellular and molecular mechanisms seem to be involved in the anti-cancer effects of HODEs. Hampel et al. [10] indicated that 9- and 13-HODE, as endogenous fatty acid ligands for PPARγ, possess differential, ligand specific actions in monocytic cells to regulate cell cycle progression, apoptosis and PPARγ2 gene expression. Interesting, recent studies show that (S)-HODE decrease cell growth and DNA synthesis, and induce apoptosis of Caco-2 cells, whereas (R)-HODE presents the opposite action [11]. Therefore, the present study considered whether 9S-HOD affects acute leukemia HL-60 cells, and what is the mechanism? The aim of this study was to investigate the effects of 9S-HOD on acute leukemia HL-60 cells. Furthermore, 9S-HOD-treated HL-60 cells were analyzed by a 2D-gel based proteomics approach and differentially expressed proteins were identified by mass spectrometry. The integration of proteomics and bioinformatics analyses revealed 9S-HOD may affect several pathways.

## Methods

### Materials

HODs (≥98.0 %, HPLC) were purchased by Larodan Fine Chemicals AB (Solna, Sweden), t10,c12-Conjugated linoleic acid (t10,c12-CLA, ≥96.0 %, HPLC) were purchased from Aldrich Chemical Co (Milwaukee, USA). RPMI 1640, CD Hybridoma, AIM-V media, Apoptosis Assay Kit#2 and Trizol LS were purchased from GIBCO (Carlsbad, USA). All reagents for 2-D electrophoresis were obtained from Amersham Pharmacia Biotech (Uppsala, Sweden).

### Cell culture and cell viability assay

HL-60 cells were obtained from the Chinese Academy of Sciences Cell Bank. They were cultured in RPMI 1640 medium plus 10 % (v/v) FBS under standard culture conditions. Prior to seeding cells to the microplates, cells media were replaced with serum (FBS)-free/ (or serum) culture media. Cells were then sub-cultured and treated with drugs which were dissolved in ethanol at a range of concentration (7.5 ~ 240 μM). After 24–48 h incubation, CCK-8 (20 μL) was added of to each well and incubated for 3 h. The final concentration of ethanol was <0.2 % (v/v). Cell viability was measured using a microplate reader (Polarstar, BMG Labtechnologies) at a wavelength of 490 nm. The effects of the drugs were expressed as percentage of cell viability against concentrations of drugs. Each performed in triplicate for three times.

### Apoptosis assay and DAPI staining

For DAPI (4′,6-Diamidino-2-Phenylindole, Dihydrochloride) staining, HL-60 cells were incubated with 9S-HOD for 48 h. The cells were collected and incubated in 0.1 mol/L PBS (pH = 7.4) containing 50 μg/ml DAPI and 100 μg/ml DNase-free RNase A at 37 °C for 20 min. Apoptotic cells were identified as typical morphology of shrinkage of the cytoplasm, membrane blebbing, and nuclear condensation and/or fragmentation. For flow cytometry analysis, cells treated with vehicle and 9S-HOD for 0–48 h were collected and stained with Annexin V-FITC and propidium iodide (PI) according to the manufacturer’s protocol and analyzed by flow cytometry, followed by data analysis using Cell Quest v.3.1 Software (Becton Dickinson, USA).

### Protein separation by 2-DE

HL-60 cells were cultured in RPMI 1640 media with or without 9S-HOD (20 μM) for 48 h. To perform 2-DE, the cells were dissolved in lysis buffer (7 M urea, 4 % CHAPS, 40 mM Tris, 65 mM DTT). Samples were sonicated on ice for 10 s with an ultrasonic processor and centrifuged for 1 h at 40,000 × g at 4 °C. Protein concentration was measured by the method of Bradford with bovine serum albumin used as the standard. Proteins (120 μg) were resuspended with 300 μl of IEF rehydration buffer (7 M urea, 2 M thiourea, 2 % CHAPS, 2 mM TBP, 0.3 % carrier ampholytes pH 3–10, and 0.002 % bromophenol blue). After sample loading, IEF gels were run at 250 V for 1 h, 500 V for 1 h, 1000 V for 1 h, 10,000 V for 3 h, 10,000 V for 65,000 V.h. After IEF, IPG strips were processed to reduction and alkylation in two equilibration steps. Strips were first equilibrated for 15 min in 6 ml of equilibration buffer I (1.5 M Tris pH 8.8, 6 M urea, 20 % glycerol, 1 % SDS, and 2 % DTT) and then equilibrated for 15 min in equilibration buffer II (1.5 M Tris pH 8.8, 6 M urea, 20 % glycerol, 1 % SDS, and 2.5 % iodoacetamide). The second dimension separation was performed with 12.5 % SDS-PAGE in Protean II xicell (Bio-Rad) and run at 10 mA for 1 h and then 28 mA for 9.5 h at 16 °C, until the dye front reached the gel border. The analytical gels were visualized with silver staining method that was mass spectrometry compatible [12]. After that, the digitalized gel images were analyzed (spot detection, count and map matching) were performed using PDquest 8.0.1 software (Bio-Rad).

### In-Gel digestion and identification

In-gel digestion was performed according to the method described by Rosenfeld [12]. The stained protein spots were excised from the gel and digested. The gel pieces were rinsed three times and spots were extracted with 25 mM NH_4_HCO_3_ and 50 % acetonitrile, and then dried completely. The dried gels were rehydrated in a digestion buffer of 25 mM NH_4_HCO_3_ containing 10 ng/μl modified trypsin. The gel slices were completely covered by the digestion buffer and left to incubate overnight at 37 °C.

### MS identification and database search

Peptide mixtures of each gel spot were dissolved in 0.1 % TFA, desalted, and concentrated. The sample was mixed with the same volume of matrix (CHCA in 30 % ACN/0.1 % TFA), spotted on a target disk, and allowed to air-dry. The peptides were analyzed using a Bruker ultrafleXtreme MALDI-TOF mass spectrometer (Bruker Daltonics, Germany). The peptide sequence was determined with MASCOT software. The search was based on the NCBI protein database on the assumption that peptides aremonoisotopic, oxidized at methionine residues, and carbamidomethylated at cysteine residues. Mass tolerance was allowed within 150 parts/million (ppm). Proteins matching more than five peptides and with a MASCOT score higher than 60 were considered significant (*P* < 0.05).

### Statistical analysis

Each assay was repeated for three times. Values were expressed as mean ± SD. Student’s t-test were used for evaluate the statistical significant differences in groups. One-way ANOVA was used to assess the differences between the vol % values (fold changes of spot volume) of each identified protein. GraphPad Prism 5 was used and *P* value of < 0.05 was considered statistically significant differences.

## Results

### The effect of FBS content in culture medium on cytotoxicity of 9S-HOD

**Fig. 1:**
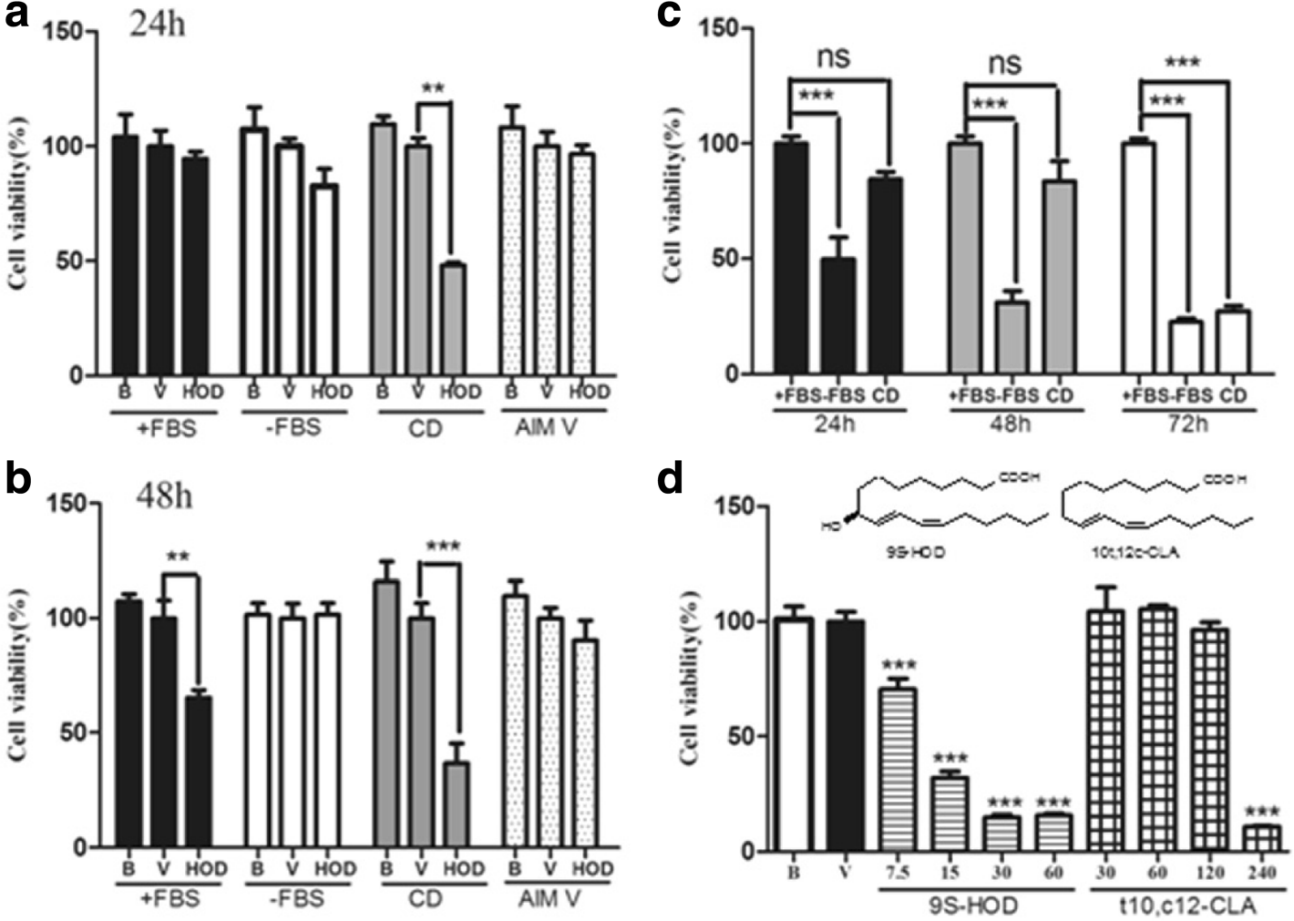
The cell viability of HL-60 cell treated with/without 9S-HOD in different culture media. **a** Effects of 9S-HOD for 24 h in + FBS, −FBS, CD and AIM-V media. **b** Effects of 9S-HOD for 48 h in + FBS, −FBS, CD and AIM-V media. **c** The cell viability of HL-60 cell for 24 ~ 72 h in + FBS, −FBS and CD media. **d** Effects of 9S-HOD or 10 t,12c-CLA for 48 h in CD medium. B: blank; V: vehicle; HOD: 9S-HOD; +FBS: RPMI medium containing 5 % FBS; −FBS: RPMI medium without FBS (serum free); CD: CD hybridoma medium (serum free); AIM-V: AIM-V medium (serum free). **P < 0.01, ***P < 0.001

The cytotoxicity of 9S-HOD in HL-60 cell was assayed by CCK-8 (Fig. 1). HL-60 cell were cultured with RPMI supplemented with 5 % FBS (+FBS), RPMI without FBS (−FBS), serum-free medium CD or AIM-V for 24–72 h. The cell viability at 24 h was markedly inhibited by 9S-HOD when cultured with CD, while 9S-HOD did not show this effect in other media (Fig. 1a). The cytotoxicity of 9S-HOD cultured in + FBS or CD media for 48 h were significantly higher than that for 24 h culture (Fig. 1b). Compared the cell viability rate in + FBS with CD media for 24 and 48 h, the cytotoxicity effect of 9S-HOD was largely attenuated by the presence of FBS in culture medium. The cell viability rate by of 9S-HOD was 49.9 % for 72 h when cultured with 5 % FBS (data not shown). However, 9S-HOD did not show cytotoxicity in –FBS or AIM-V media.

HL-60 cells were cultured in + FBS, −FBS or CD media to test cell growth (Fig. 1c). With + FBS in culture medium, the cell viability rate was used as control (100 %). There was no significant difference in the cell viability rate between + FBS and CD for 24 and 48 h (*P* > 0.05, but sharply decreased to 27.2 % for 72 h in CD culture medium (*P* < 0.05). Meanwhile, there was significant difference in the cell viability rate between + FBS and –FBS (*P* < 0.05). In FBS-free culture medium, the cell viability rates were 49.7, 30.9 and 22.4 % for 24, 48 and 72 h, respectively.

When cultured in CD medium for 48 h in the presence of 9S-HOD or 10 t,12c-CLA shown concentration-dependant cytotoxicity by the reduction of CCK-8 (Fig. 1d). 10 t,12c-CLA started to show noticeable activity at 240 μM while 9S-HOD shown significant cytotoxicity effect from 7.5 to 60 μM (*P* < 0.05).

**Fig. 2:**
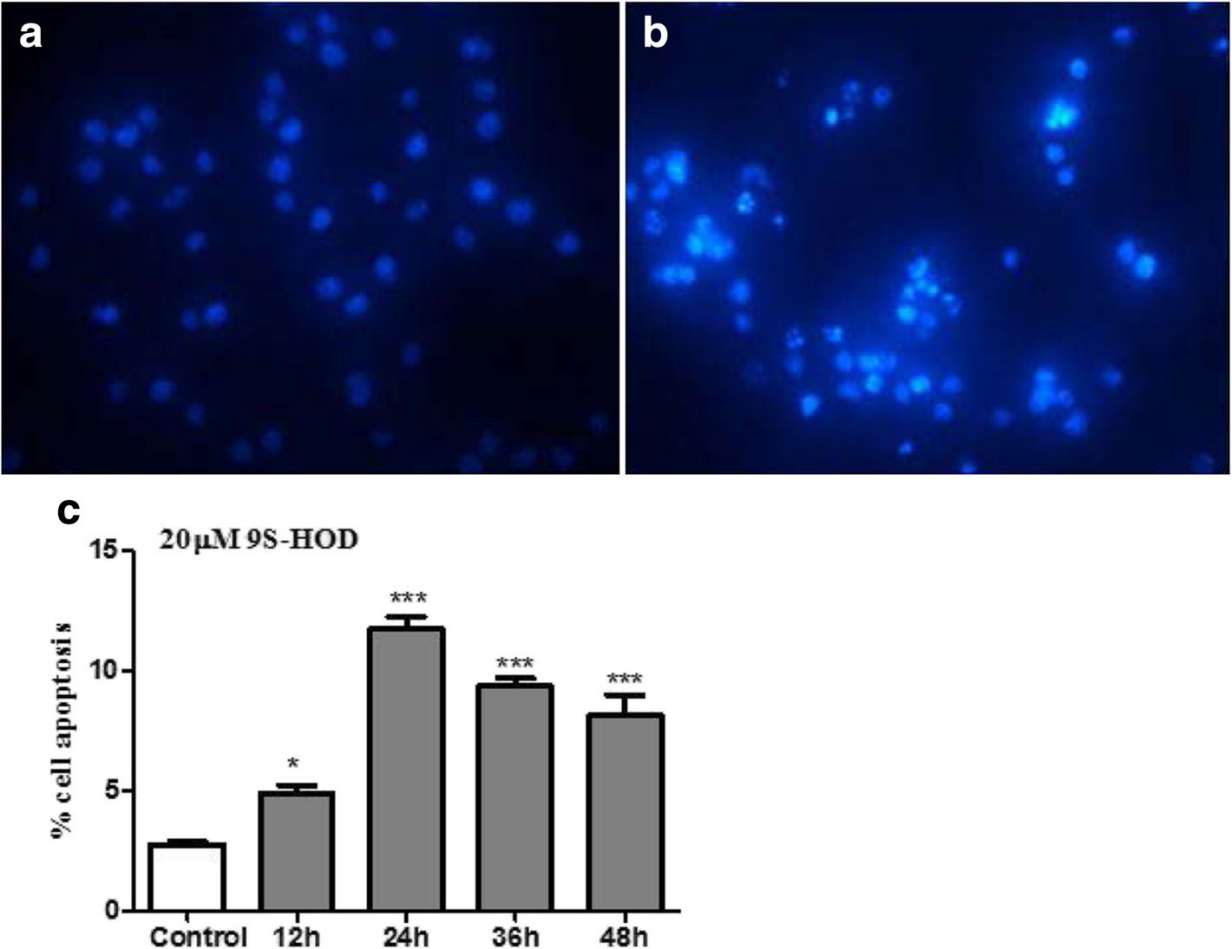
9S-HOD induces HL-60 cell apoptosis. Morphological analysis of HL-60 cell after **a** vehicle or **b** 20 μM 9S-HOD treatment for 48 h, and **c** apoptosis examined using Annexin V/PI staining and flow cytometry. *P < 0.05, ***P < 0.001

To determine whether the cytotoxicity effect was caused by cell apoptosis, HL-60 cells were treated with 20 μM of 9S-HOD for 48 h and DAPI staining was performed. Treated cells showed marked nuclear fragmentation and chromatin condensation which were clear indications of apoptosis (Fig. 2b), while control cells showed intact cell bodies with clear round nuclei (Fig. 2a). Meanwhile, we found a time-dependent increase in cell apoptosis (FITC-Annexin-V and PI double-positive cells) for 0–24 h after treated with 9S-HOD (*P* < 0.05), then the percentage of apoptosis cell was decreased from 24 to 48 h due to the late apoptosis cell increasing (Fig. 2c). Our data suggest that the cytotoxicity effect of 9S-HOD may be associated with an increase in cell apoptosis.

### Proteomic results and statistical analysis

**Fig. 3:**
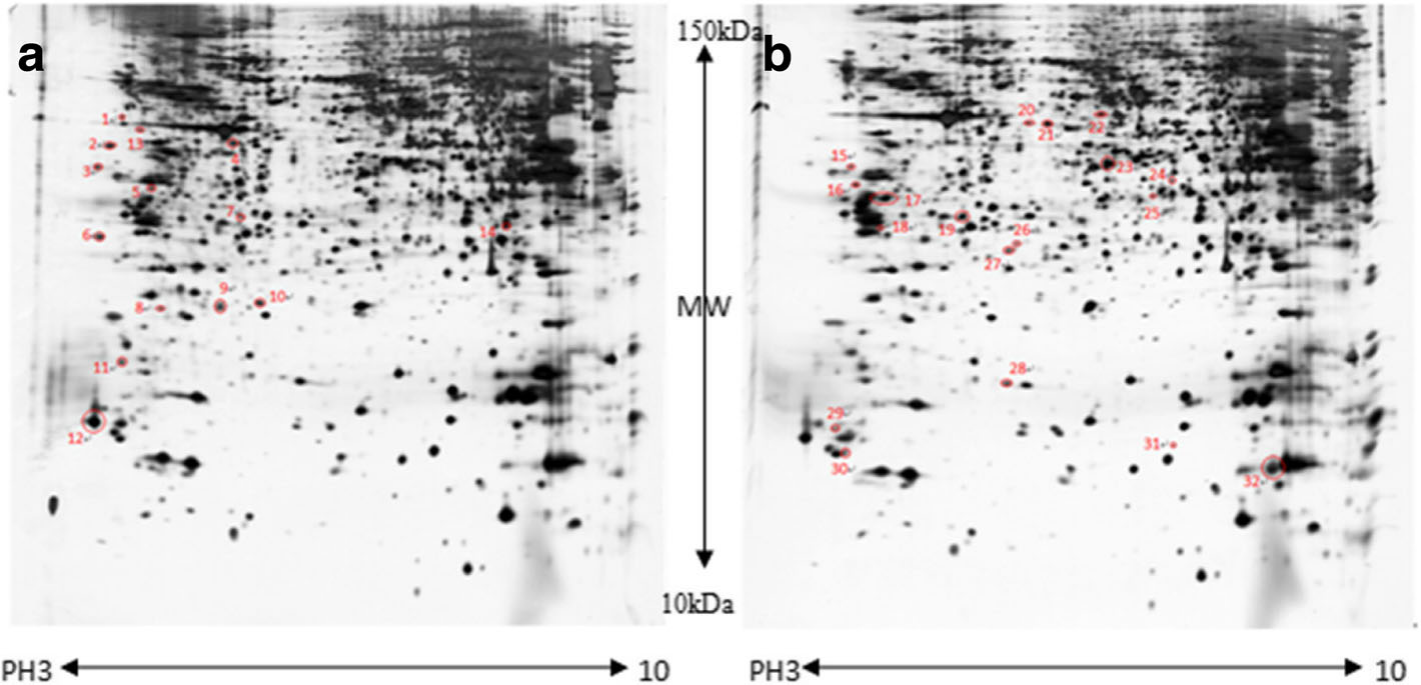
Two-DE profiles of **a** vehicle or **b** 20 μM 9S-HOD treatment of HL-60 for 48 h. Total protein exacts were separated on pH 3–10, 17 cm IPG strips and stained with silver. MS-based identification of these spots is summarized in Table 1

To investigate the mechanism, the protein expression changes upon 9S-HOD treatment in HL-60 cells were analyzed by 2-DE-based proteomics approach. After 20 μM 9S-HOD or vehicle treatment for 24 h, protein lysates were prepared and separated first by IEF followed by SDS-PAGE separation; and the resulting 2-DE gel were stained with collocidal coomassie blue silver. Then the stained 2D-gel were scanned and analyzed by GE software, 3-fold differentially expressed proteins were excised and processed for further MS analysis. In Fig. 3, representative 2-DE gels of untreated (a) and 9S-HOD treated HL-60 cell (b) are shown. As a result, the 2-DE gel showed an obvious separation of cell proteins, which 1200 protein spots were detected in each gel. Comparing the spots of untreated and 9S-HOD treated HL-60 cell by PDquest software for image analysis, a total of 32 protein spots exhibited 3-fold significant difference (*P* < 0.05). Among the 32 proteins, 20 peptide mass fingerprints (PMFs) were matched identified by MALDI-TOF-MS and protein database searching, as displayed in Table 1.

**Table 1:** The identification of protein spots on 2-DE

Spot	Accession	Protein name	Gene	M.W.	pI	Score
mRNA metabolic process						
13	Q69YN4	Protein virilizer homolog↓	KIAA1429	202584.08	4.44	66.6
9	Q13185	Chromobox protein homolog 3↓	CBX3	20969.41	4.96	84.6
22	P61978	Heterogeneous nuclear ribonucleoprotein K↑	HNRNPK	48760.25	5.17	54.5
Protein binding						
32	P60709	Actin, cytoplasmic 1 ↑	ACTB	42051.86	5.15	122
23	P05388	60S acidic ribosomal protein P0↑	RPLP0	34388.88	5.77	170
29	P11171	Protein 4.1↑	EPB41	81755.32	5.54	61
6	Q6PK50	HSP90AB1 protein↓	HSP90AB1	40270.42	4.61	78.6
DNA replication						
1	Q01105	Protein SET↓	SET	30974.22	3.86	98
2	Q01105	Protein SET↓	SET	31310.48	3.83	202
5	P12004	Proliferating cell nuclear antigen↓	PCNA	29092.42	4.31	94.1
Cytoskeleton						
21	P63261	Actin, cytoplasmic 2 ↑	ACTG1	42107.92	5.16	321
17	I3L1U9	Actin, cytoplasmic 2, N-terminally processed↑	ACTG1	23886.98	5.11	129
Apoptosis process						
26	P51878	Caspase-5↑	CASP5	33966.33	8.55	60.4
Negative regulation of myeloid cell differentiation						
4	E9PKG1	Protein arginine N-methyltransferase 1↓	PRMT1	38198.03	6.06	106
Unknown						
8	P35527	Keratin, type I cytoskeletal 9↓	KRT9	62254.9	4.89	67.1
31	Q15195	Plasminogen-like protein A↑	PLGLA	11135.6	6.5	63.6
28	Q8IUZ0	Isoform 3 of Leucine-rich repeat-containing protein↑	LRRC49	74491.59	7.15	60
27	B4DVQ0	cDNA FLJ58286, highly similar to Actin, cytoplasmic 2↑		37666.76	5.45	147
20	B4E335	cDNA FLJ52842, highly similar to Actin, cytoplasmic 1↑		39542.66	5.28	207
19	B4DW52	cDNA FLJ55253, highly similar to Actin, cytoplasmic 1↑		38950.3	5	148

### Bioinformatic analysis

**Fig. 4:**
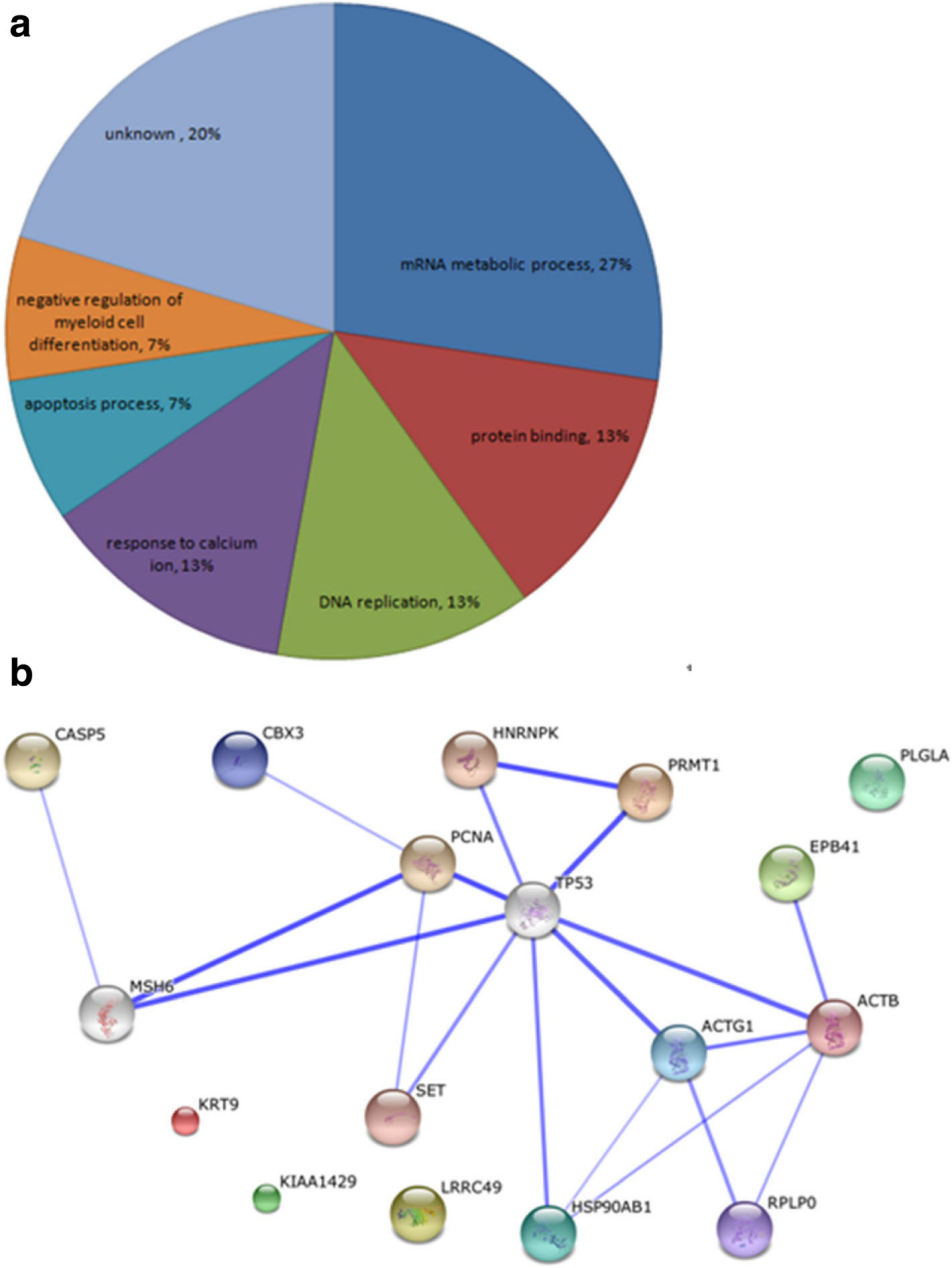
Distribution of GO annotations of biological functions for identified proteins. **a** Multi-level pie chart of combined graph of GO biological functions annotations by Genecodis2; **b** The network of proteins was generated by STRING

We analyzed the GO enrichment of fourteen up-regulated and six down-regulated proteins in 9S-HOD treated HL-60 cells (Table 1) by using the http://genecodis.cnb.csic.es/ website. These dysregulated proteins were categorized into different biology processes including response to mRNA metabolic process, protein binding, DNA replication and apoptosis (Fig. 4a). STRING software was used to map a network of interacting proteins whose levels changed following 9S-HOD treatment.

## Discussion

It is reported that HODs showed more potent anti-tumor activities than lead compound CLA, especially (S)-HOD had more activity compared with the corresponding (R)-HOD [11, 13]. To date, the mechanism of 9-HOD involved of inhibiting cell proliferation and inducing apoptosis. In this study, we also observed cytotoxicity and apoptosis in HL-60 cells after treatment of 9S-HOD, observing typical morphological changes such as chromatin condensation and fragmentation. Furthermore, the 9S-HOD anti-tumor activity on HL-60 cell was severely delayed and attenuated by the presence of FBS in culture medium, which was correspond well to our previous finding on other cancer cells [14]. This may be due to serum albumin in culture medium could strongly bind to fatty acid [11]. Interestingly, in the present study, CD media (serum-free) were demonstration to increase the antitumor activity of 9S-HOD, whereas the activity were weak in –FBS and AIM V media (also serum-free). This may be due to the HL-60 cells grew not well in –FBS media with the cell viability of 49.7 % for 24 h, 30.9 % for 48 h compared to that in FBS media as 100 %.

Our proteomic study revealed differences between control and 9S-HOD treated group. 33 differentially expression proteins were found, 19 of which were successfully identified by mass spectrometry followed by NCBI database. Functional annotation analysis showed that the differential proteins were involved in various biological processes such as mRNA metabolic process, protein binding, DNA replication, response to calcium ion, apoptosis and negative regulation of myeloid cell differentiation.

In order to better understand the mechanisms of 9S-HOD in HL-60, the interaction network of proteins was built by the STRING as described previously shown as Fig. 4b. Most of the proteins in the map have direct or indirect links, in which protein ACTB, EPB41, HSP90AB1 and RPLP0 functioning in protein folding and processing form a small close network, suggesting that 9S-HOD may regulate this pathway. Among these proteins, hot shock protein 90 (Hsp90) is associated with tumorigenesis. The availability of drugs that can specifically target Hsp90 and inhibit its function, resulting in the depletion of client proteins, has made Hsp90 a novel and exciting target for the development of anti-tumor drugs [15]. Hsp90 has two main isoforms, namely, Hsp90-alpha (Hsp90AA1) and Hsp90-beta (Hsp90AB1) [16]. A study of various tumor cell lines revealed that Hsp90-beta was up-regulated expression in A549, H520, H446 and HL-60 cell lines [17]. In our results, we detected the remarkably 4-fold down-regulated expression of Hsp90AB1 in the 9S-HOD treated group, implicating that 9S-HOD may affect on the protein folding and thus inhibited cell proliferation. Meanwhile, EPB41 with functioning in positive regulation of protein binding was up-regulated by 9S-HOD treatment.

On 2-DE, PCNA is usually detected as a single spot, as previously described [18]. The exactly PCNA theoretical parameters is pI ~4.57 and mass ~30 kDa on the 2-DE map. The PCNA spot in this study corresponded to a polypeptide of pI 4.31 and mass 29 kDa, showing that the PCNA is not post-translational modified [19]. It is also interesting to note that PNCA protein was the core node associated with many other proteins in the network. PCNA is the key cell cycle marker protein, involving in DNA replication, DNA repair, chromatin remodeling and epigenetics [20, 21]. Interaction with different protein partners is an important regulatory mechanism for the diverse functions of PCNA. Since more than 100 PCNA-interacting proteins and several PCNA modifications have already been reported [22]. Among the proteins involved in protein processing, EPB41 and Hsp90AB1 were interactive with PCNA in this study, which also has been verified previously [23, 24]. It is reported that PCNA was one of the Hsp90 client proteins in HCT-116 [24], suggesting that the association of PCNA with Hsp90 might be a general phenomenon in cancer cells. The same phenomenon was found in our results that Hsp90AB1 and PCNA protein were down-regulated by 9S-HOD in HL-60.

Another interesting protein is the 60s acidic ribosomal protein p0 (RPLP0), which belongs to the L10P family of ribosomal proteins. Proteomic profiling pointed to an association between RPLP0 up-regulation and cancer in several tumor cells [25], which is involved in mRNA translation. In addition, a recent report showed that RPLP0 was up-regulated in patients with the most common types of cancer, indicating that the expression level of RPLP0 could be useful as a prognostic marker in tumors [26]. In our study, 9S-HOD caused down-regulated of RPLP0 protein in HL-60 cells.

Three proteins had been demonstrated to be related with mRNA metabolic process, including KIAA1429, CBX3 and hnRNPK. RNA process is important for the proliferation of tumor cells and RNAi knock-down of RNA processing proteins in cancer cells induces apoptosis [27]. In agreement with these findings, the protein hnRNPK was significantly down-regulated by 9S-HOD. hnRNPK is a nucleic acid-binding protein that serves as a docking platform integrating transduction pathways to nucleic acid-directed processes [28]. Knock-down of hnRNPK results in strongly reduced migratory activity of cancer cells, strongly suggesting hnRNPK as a promising target molecule for cancer progression [29].

## Conclusion

In conclusion, 9S-HOD exerted cytotoxicity efficacy and induced apoptosis in HL-60 cells, and the cytotoxicity was largely attenuated by the presence of FBS in culture media. A classical proteomic approach is performed to elucidate the mechanism of its anti-leukemia activity by 9S-HOD. Based on 2-DE and MALDI-TOF-MS, 23 differentially expressed proteins were identified. Bioinformatics analysis on the proteomic alterations suggested that 9S-HOD may affect several pathways such as mRNA metabolic process, protein binding, DNA replication and apoptosis. However, further studies would be necessary to clarify the exact targets by 9S-HOD in treating leukemia.

## Abbreviations

13-HOD: 13-hydroxy-octadecadienoic acid
15-LOX: 15-lipoxygenase
2-DE: Two-dimensional gel electrophoresis
9-HOD: 9-hydroxy-octadecadienoic acid
9S-HOD: 9s-hydroxy-octadecadienoic acid
AML: Acute myeloid leukemia
DAPI: Dihydrochloride
FBS: Fetal bovine serum
HODEs: Hydroxyl-octadecadienoic acids
MS: Mass spectrometry
PI: Propidium iodide
PMFs: Peptide mass fingerprints
t10,c12-CLA: t10,c12-Conjugated linoleic acid

## References

[1] Lindsley RC, Mar BG, Mazzola E, Grauman PV, Shareef S, Allen SL (2015). Acute myeloid leukemia ontogeny is defined by distinct somatic mutations. Blood.

[2] Bahar MA, Andoh T, Ogura K, Hayakawa Y, Saiki I, Kuraishi Y (2013). Herbal medicine goshajinkigan prevents paclitaxel-induced mechanical allodynia without impairing antitumor activity of paclitaxel. Evid Based Complement Alternat Med.

[3] Buchi F, Pastorelli R, Ferrari G, Spinelli E, Gozzini A, Sassolini F (2011). Acetylome and phosphoproteome modifications in imatinib resistant chronic myeloid leukaemia cells treated with valproic acid. Leuk Res.

[4] Mosblech A, Feussner I, Heilmann I (2009). Oxylipins: structurally diverse metabolites from fatty acid oxidation. Plant Physiol Biochem.

[5] Simsek S, Doehlert DC (2014). Oxygenated fatty acids isolated from wheat bran slurries. Int J Food Sci Nutr.

[6] Ruparel S, Green D, Chen P, Hargreaves KM (2012). The cytochrome P450 inhibitor, ketoconazole, inhibits oxidized linoleic acid metabolite-mediated peripheral inflammatory pain. Mol Pain.

[7] Chang J, Jiang L, Wang Y, Yao B, Yang S, Zhang B (2015). 12/15 Lipoxygenase regulation of colorectal tumorigenesis is determined by the relative tumor levels of its metabolite 12-HETE and 13-HODE in animal models. Oncotarget.

[8] Yuan H, Li MY, Ma LT, Hsin MK, Mok TS, Underwood MJ (2010). 15-Lipoxygenases and its metabolites 15(S)-HETE and 13(S)-HODE in the development of non-small cell lung cancer. Thorax.

[9] Pasqualini ME, Heyd VL, Manzo P, Eynard AR (2003). Association between E-cadherin expression by human colon, bladder and breast cancer cells and the 13-HODE:15-HETE ratio. A possible role of their metastatic potential. Prostaglandins Leukot Essent Fatty Acids.

[10] Hampel JKA, Brownrigg LM, Vignarajah D, Croft KD, Dharmarajan AM, Bentel JM (2006). Differential modulation of cell cycle, apoptosis and PPARγ2 gene expression by PPARγ agonists ciglitazone and 9-hydroxyoctadecadienoic acid in monocytic cells. Prostaglandins Leukot Essent Fatty Acids.

[11] Cabral M, Martin-Venegas R, Moreno JJ (2014). Differential cell growth/apoptosis behavior of 13-hydroxyoctadecadienoic acid enantiomers in a colorectal cancer cell line. Am J Physiol Gastrointest Liver Physiol.

[12] Rosenfeld J, Capdevielle J, Guillemot JC, Ferrara P. In-gel digestion of proteins for internal sequence analysis after one- or two-dimensional gel electrophoresis.Anal Biochem.1992;203:173-9. 10.1016/0003-2697(92)90061-b 1524213

[13] Vangaveti VN, Shashidhar VM, Rush C, Malabu UH, Rasalam RR, Collier F (2014). Hydroxyoctadecadienoic acids regulate apoptosis in human THP-1 cells in a PPARγ-dependent manner. Lipids.

[14] Li Z, Tran VH, Duke RK, Ng MC, Yang D, Duke CC (2009). Synthesis and biological activity of hydroxylated derivatives of linoleic acid and conjugated linoleic acids. Chem Phys Lipids.

[15] Richardson PG, Mitsiades CS, Laubach JP, Lonial S, Chanan-Khan AA, Anderson KC (2011). Inhibition of heat shock protein 90 (HSP90) as a therapeutic strategy for the treatment of myeloma and other cancers. Br J Haematol.

[16] Langer T, Fasold H (2001). Isolation and quantification of the heat shock protein 90 alpha and beta isoforms from rat liver. Protoplasma.

[17] Biaoxue R, Xiling J, Shuanying Y, Wei Z, Xiguang C, Jinsui W (2012). Upregulation of Hsp90-beta and annexin A1 correlates with poor survival and lymphatic metastasis in lung cancer patients. J Exp Clin Cancer Res.

[18] Jiang N, Kham SK, Koh GS, Suang Lim JY, Ariffin H, Chew FT, et al. Identification of prognostic protein biomarkers in childhood acute lymphoblastic leukemia (ALL). J Proteomics. 2011;74(6):843-57. 10.1016/j.jprot.2011.02.034 21396490

[19] Naryzhny SN, Lee H (2007). Characterization of proliferating cell nuclear antigen (PCNA) isoforms in normal and cancer cells: there is no cancer-associated form of PCNA. FEBS Lett.

[20] Miller A, Chen J, Takasuka TE, Jacobi JL, Kaufman PD, Irudayaraj JM (2010). Proliferating cell nuclear antigen (PCNA) is required for cell cycle-regulated silent chromatin on replicated and nonreplicated genes. J Biol Chem.

[21] Strzalka W, Ziemienowicz A (2011). Proliferating cell nuclear antigen (PCNA): a key factor in DNA replication and cell cycle regulation. Ann Bot.

[22] Naryzhny SN (2008). Proliferating cell nuclear antigen: a proteomics view. Cell Mol Life Sci.

[23] Ewing RM, Chu P, Elisma F, Li H, Taylor P, Climie S (2007). Large-scale mapping of human protein-protein interactions by mass spectrometry. Mol Syst Biol.

[24] Wang X, Heuvelman DM, Carroll JA, Dufield DR, Masferrer JL (2010). Geldanamycin-induced PCNA degradation in isolated Hsp90 complex from cancer cells. Cancer Invest.

[25] Frozza CO, Ribeiro TS, Gambato G, Menti C, Moura S, Pinto PM (2014). Proteomic analysis identifies differentially expressed proteins after red propolis treatment in Hep-2 cells. Food Chem Toxicol.

[26] Artero-Castro A, Castellvi J, Garcia A, Hernandez J, Ramon YCS, Lleonart ME (2011). Expression of the ribosomal proteins Rplp0, Rplp1, and Rplp2 in gynecologic tumors. Hum Pathol.

[27] Naoghare PK, Tak YK, Kim MJ, Han E, Song JM (2011). Knock-down of argonaute 2 (AGO2) induces apoptosis in myeloid leukaemia cells and inhibits siRNA-mediated silencing of transfected oncogenes in HEK-293 cells. Basic Clin Pharmacol Toxicol.

[28] Barboro P, Ferrari N, Balbi C (2014). Emerging roles of heterogeneous nuclear ribonucleoprotein K (hnRNP K) in cancer progression. Cancer Lett.

[29] Marques HM, Simpson D, Ngok SP, Nieves B, Chen R, Siprashvili Z (2014). Long non-coding RNA EWSAT1-mediated gene repression facilitates Ewing sarcoma oncogenesis. J Clin Invest.

